# RONI Based Secured and Authenticated Indexing of Lung CT Images

**DOI:** 10.1155/2015/830453

**Published:** 2015-05-20

**Authors:** I. Jasmine Selvakumari Jeya, J. Suganthi

**Affiliations:** Department of Computer Science and Engineering, Hindusthan College of Engineering and Technology, Coimbatore, Tamil Nadu 641 032, India

## Abstract

Medical images need to be transmitted with the patient's information without altering the image data. The present paper discusses secured indexing of lung CT image (SILI) which is a secured way of indexing the lung CT images with the patient information. Authentication is provided using the sender's logo information and the secret key is used for embedding the watermark into the host image. Watermark is embedded into the region of Noninterest (RONI) of the lung CT image. RONI is identified by segmenting the lung tissue from the CT scan image. The experimental results show that the proposed approach is robust against unauthorized access, noise, blurring, and intensity based attacks.

## 1. Introduction 

The process of hiding information into a media file is called watermarking. The hidden information shall be an image or a string and the host image shall be images, audio, or video files. The principal purpose behind watermarking is to resolve copyright issues. Logo insertion is the popular method used in watermarking. In this, logo is inserted into the respected image during watermarking to avoid intellectual property disputes. This process becomes more secure by using a security key in watermarking. The secret key should be known only to the authorized sender and receiver. Extraction process retrieves back the watermark and the host using the secret key.

Digital image watermarking is the process of embedding the watermark into digital images. This embedding can be done using various domains-frequency domain and spatial domain. Applications other than copyright use an invisible watermarking technique. However, during the process of watermarking medical images, care should be taken to preserve the important patient information present in the image. The watermarked image sent to the receiver should provide information about the sender. This authentication protects from unauthorized access over the watermarked image. When patient's information is embedded into the images and transmitted, transmission time and cost gets greatly reduced.

### 1.1. Watermarking

Watermarking is performed in two ways, irreversible watermarking and reversible watermarking. Irreversible watermarking is the process of permanently embedding watermark image into the host image. Therefore, this approach greatly helps in providing copyrights to images. Here, the watermarks used are either logo or the name of the image' owner. Generally irreversible watermarks are visible to the viewers. Barton initiated the process of reversible watermarking in 1997 [1] which compressed the watermark bits and embedded to the data blocks of the host. Pakdaman et al. [2] proposed a reversible blind watermarking technique based on the Hadamard transform. The prediction error coefficients were used to embed the watermark into the host. Using this method, both the original and the watermark images can be retrieved back without the help of a location map. It also increases the embedding capacity.

The pixel values of the watermark and the host are often used to embed the watermark into the host. Honsinger et al. [3] used the pixel values of the watermarked image to retrieve back the original image in a lossless way. A histogram based approach [4] involved embedding of watermark based on the values obtained from the histogram. Lin et al. [5] proposed multilevel watermarking to overcome the drawbacks of low embedding capacity watermarking [4]. A preliminary step before computation of the histogram helped in increasing the embedding capacity of the algorithm. In some methods, adjacent pixels were initially used to get the region of embedding [6–8].

Zain and Clarke [9] had proposed a watermarking algorithm to retrieve back the original image from the watermarked image. Secure Hash Algorithm 256 (SHA 256) was applied in embedding and extraction. The image can be retrieved only when the image has not gone through any attacks. Simple embedding and extracting of watermarks from the original image has been ruled out by security enabled watermarking schemes. Unauthorized access and tampering of images are overcome by using these methods. Houmansadr and Ghaemmaghami [10] proposed an image watermarking scheme which used visual cryptography to embed the watermark image. Here, the watermark image was divided into two parts: one part embedded as a watermark image and another part used as a secret key to provide security. Only an authorized person, who knows the secret key, will be able to retrieve back the watermark and other details. Anand and Niranjan [11] proposed a method to embed the measured physiological signals into the images before transmission for successful retrieval at the other end.

Medical images need special attention at the region of interest (ROI) regions. ROI is different for different medical images. Based on the source of medical image, ROI is identified and further processing needs to be done. Methods proposed in literature [12–14] avoid ROI for embedding the watermark image. The main reason for avoiding embedding of watermarks in ROI is to preserve the image quality. Memon et al. [15] used both ROI and RONI for multiple watermarking on medical images. Initially, RONI is used to provide security, authentication, and confidentiality. Later, ROI is identified and a copy is made by embedding it outside ROI to get a distortion less image later.

While embedding watermarks into the lung CT images, the lung lobes need to be initially segmented. The lung lobes represent ROI and the remaining portions are RONI. Several segmentation methods can be used to segment ROI from lung CT images. Fuzzy C-Means were used by Karthikeyan et al. [16] to segment the lung tissue followed by noise, airways removal, and morphological operations. A region based approach was proposed by Zhou et al. [17] to segment the lungs. The input CT scan image was given to the sequential splitting process to get the segmented lung lobes. A dynamic programming algorithm was also used to segment lung tissues from the background information of lung CT scan image from a sequence of CT images [18].

### 1.2. Indexing

The database of hospitals and clinical labs is voluminous which makes storage and retrieval highly expensive. Watermarking can also be used to index the medical images. While indexing these databases with the relevant data, the storage bandwidth is greatly reduced and thereby making retrieval easier. There is hardly any proven method which shows better performance in both embedding and extraction of watermarks in medical images. Though very few works have been carried out to use watermarking for indexing, it is very useful for easy retrieval. Cheng et al. [19] discusses two different nonubiquitous digital watermarking methods to provide indexing of medical images. Here, the embedding of medical information record is done in RONI. An image hash function [20] is also proposed to index the medical images. Message authentication codes (MACs) were used in such a way that no two hash values collide.

Watermarking finds various applications over different images, especially medical images. Based on the watermark used, the watermarking procedure can be used for various applications. Robust watermarks [21] shall be used for copyright applications. Data authentication applications need watermarks which can be easily retrieved back and modified. Fragile watermarks [22, 23] help these applications. Watermarking is used for indexing purposes which may help in easy storage and retrieval of patient's details.

In the present paper, the proposed watermarking scheme uses as a logo image in a watermark with a secret key. The patient's information is embedded into lung CT images, thus providing a method for indexing the images by the sender. The receiver can extract the information from the watermarked image only when the secret key is revealed. The secret key is further made more secure with the help of an encryption algorithm. The robust watermarking procedure is explained in the following section.

## 2. Materials and Methods

### 2.1. Overview of SILI

**Figure 1:**
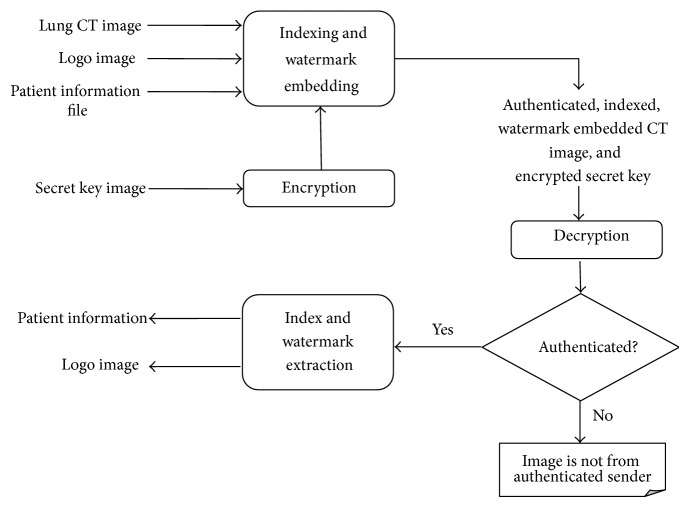
Overall block diagram of SILI.

SILI aims at indexing authenticated and watermark embedded lung CT images. At the sender side, the lung CT image is indexed using a secured way and double embedding is done. A secret key is initially encrypted using data encryption standard (DES) algorithm [23]. This encrypted secret key is used in embedding the patient details and logo image into the lung CT image. Double embedding is done over the indexed image using the patient information and logo image. The patient information is stored in a file and the logo image is separately given. The logo image is watermarked into the RONI identified. Embedding patient's information into the host lung CT image helps in indexing the medical dataset. It also helps in reducing the bandwidth used for sending separate information file. Embedding logo image assists in authentication of the sender. This authenticated, indexed, and watermark embedded lung CT image is sent to the receiver who already possesses the identity of the sender. At the receiver's side, the transmitted encrypted secret key is decrypted to find the secret key. After successful authentication, the watermark image, that is, logo image, and patient information are successively extracted.  Figure 1 shows the overall block diagram of SILI.

#### 2.1.1. SILI at Sender

**Figure 2:**
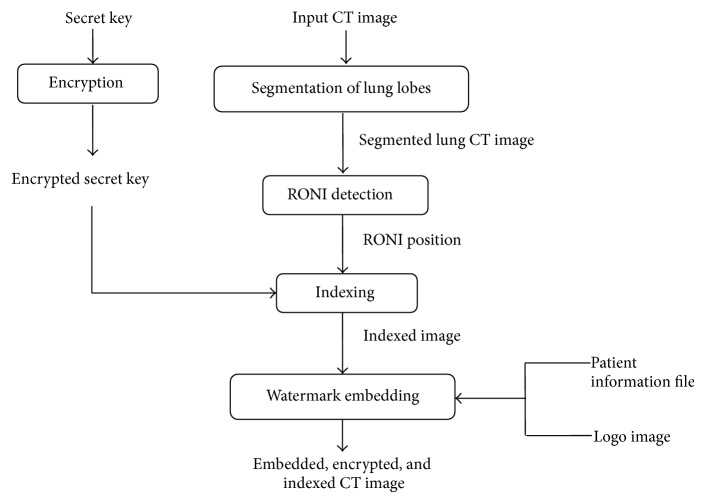
Watermark embedding and authentication at the sender side.

Figure 2 shows the steps carried out at the sender's side before sending the lung CT image to the receiver. The lung CT image, logo of the sender, patient information file, and a secret key are given as input for SILI at the sender's side. The patient information file serves as index which helps in indexing the input lung CT images. To provide secured and reliable indexing, the secret key is encrypted using DES algorithm and used in secured watermarking of the logo image.

The patient's details obtained from the input CT image are stored into “patient information details” file. The logo image of the sender is provided externally for authentication. Watermark embedding is done with intense care such that it does not affect the important portion of the medical image. RONI detection helps in preserving the main portion of the image and identifying the non-ROI portions from the lung CT image. Logo embedding is done over the indexed lung CT image. It is carried out at the identified RONI portion. Finally, watermark embedded indexed lung CT image is transmitted to the receiver.

#### 2.1.2. SILI at Receiver

The first step carried out at the receiver side is authentication. It helps in verifying whether the received image has been sent by an authenticated server. Here, secret key is employed to check the sender's true identity. The lung CT image received from the sender is initially given to a process of index extraction. This process retrieves the encrypted secret key. It is then decrypted to find the original secret key used by the sender. The decrypted secret key is compared with the original key applied at the sender side. If the secret key fails to verify the sender, the process stops. If the obtained secret key verifies the authentication of the sender, the remaining tasks of extracting the watermarks are carried out.

**Figure 3:**
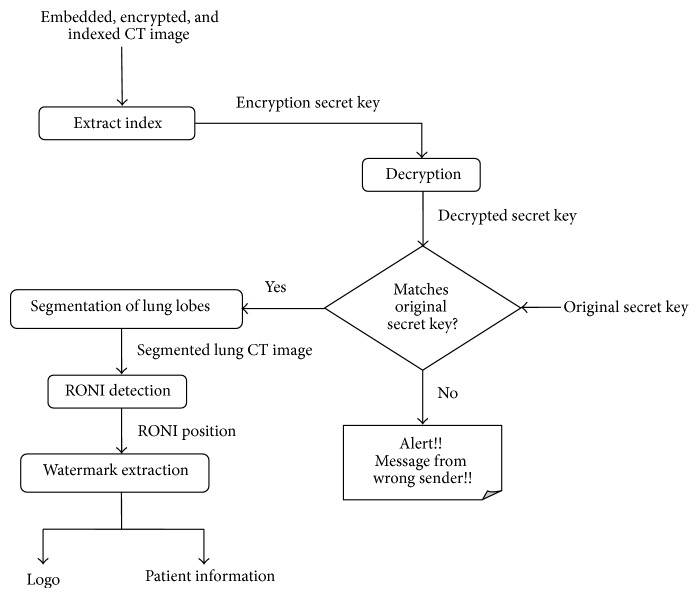
Authentication verification and watermark extraction at the receiver side.

The initial step in the extraction of watermarks is to find RONI from the lung CT image. Hence, segmentation of lung lobes and RONI detection takes place. Using the obtained RONI position, watermark extraction algorithm is applied to recover back the logo image and patient information. Extraction of logo image helps in verifying the sender's identity and the patient information obtained that can be used to keep record of the patient's details.  Figure 3 depicts the activities executed to get back the patient's details and the index from the embedded, encrypted, and indexed lung CT image.

### 2.2. RONI Based Secured and Authenticated Indexing

#### 2.2.1. Region of Noninterest (RONI) Detection

RONI is the portion in the image whose distortion does not affect the medical image's essential data. Embedding the watermark into such region helps in preserving the actual data present in the medical image. Lung CT images are mainly used for detecting the lung nodules thus assisting the detection of the presence of lung cancer. The lung lobes which may possess the lung nodules are the most important region in the CT scan. These lung lobes occupy a larger portion. In addition to lung lobes, other unnecessary portions are also scanned during the process. Those regions act as RONI. In lung CT images, the portion between the lobes and the regions surrounding the lobes are taken as RONI and the successive embedding of watermark is carried out.

**Figure 4:**
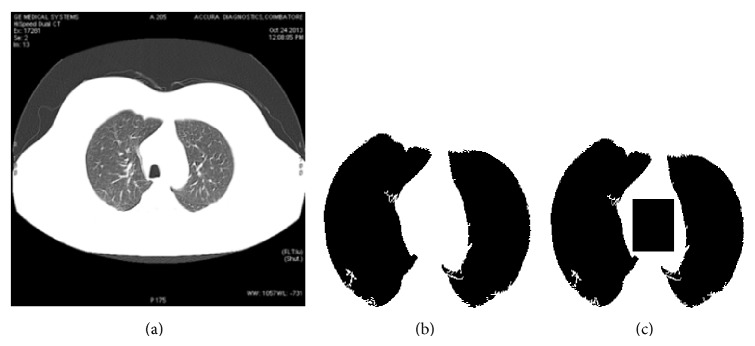
(a) Input image. (b) Segmented lobes. (c) RONI detected portion between lobes.

To detect RONI from the lung CT images, lung lobes need to be segmented and preserved such that watermark embedding does not affect them. From the input image, lung tissue is separated using a thresholding procedure. The isolated lung tissue has some unwanted portions attached to its edges, called artifacts. These artifacts are removed using the borders of the separated lung lobes. Later, the exact lobes are extracted and segmented. After successful segmentation, RONI is determined. The portion between the lung lobes is chosen to embed the logo image of the sender. With the information of the image's width and height and the position of the segmented lobes, the area between the lobes is identified and retrieved for logo embedding. The procedure for RONI detection is given in Algorithm 1. The input and result of the algorithm are shown in Figures 4(a), 4(b), and 4(c), respectively.

#### 2.2.2. Encryption and Decryption

The key used for watermark embedding is initially encrypted using DES algorithm. Here, the scan number and the image number are used as plain text and a key is provided by the sender. DES algorithm helps in converting this plain text into secret key. After 16 rounds of functions, the plain text is converted into cipher text. The secret key is now in an encrypted format such that the others cannot understand the actual meaning of the message. This encrypted secret key is used in secured watermark embedding of the logo image. This double check procedure greatly improves the authentication procedure to verify the identity of the sender.

#### 2.2.3. Indexing and Index Extraction

Each image has its own patient details and is retrieved from the patient information file. Indexing of patient information into the scan image (refer Algorithm 2) is done using the character cap. Reverse process is performed to extract the embedded information from the image. Initially, the host image is converted and represented in a single column matrix. The length of patient information is found and the image's capability to accommodate it is verified. The following condition is used for verification:

(1) $$\begin{array}{c} {\text{Size}}_{\text{host}}\geq \left(\;\text{NCh}+1\;\right)\times 8, \end{array}$$

where NCh is the number of characters present in the patient's information. If the image can accommodate the given string, the index of the character's bit to be altered is then calculated. The bit is accordingly altered to insert the string into the image. Finally, a character cap is inserted to indicate the end of the string. Extracting the patient information at the receiver side is done by exactly reversing the procedure of indexing done at the sender's side. A flag is used to find the character cap. It is set if the procedure finds the end of the string; else the string is retrieved from the bits of the image and finally the engraved patient information is retrieved from the lung CT image.

#### 2.2.4. Embedding and Extraction of Watermark

Instead of embedding the watermark image into the entire host image, a portion is detected as RONI and the watermark is embedded only into the detected portion. Embedding and extraction takes place in the presence of a key. This means, if a different key is used for extraction other than the key used during embedding; the extraction process stops and gives alert. The key used here is the encrypted secret key. Watermarking is the process of embedding the logo image into the host image, that is, lung CT image's RONI portion in a secured manner using a key. This secured region based watermark embedding algorithm is given in Algorithm 3. The input to this algorithm is the host image, index position of RONI, secret key, and the watermark image. The lung CT image acts as host image and the identified RONI portion provides the index position. The result of DES algorithm provides the encrypted secret key and it helps in providing security to the embedding and extraction procedure. Here, the logo image is the watermark image.

**Figure 5:**
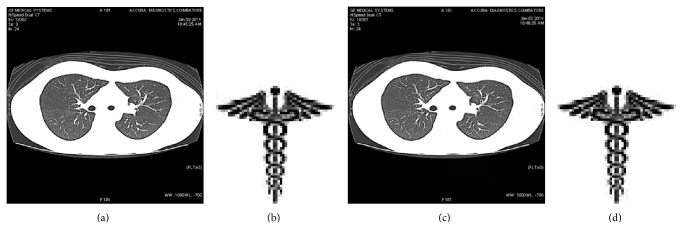
(a) Input lung CT image. (b) Logo image. (c) Watermarked lung CT image. (d) Retrieved logo image.

The preliminary check is to verify whether the host image can be used to embed the watermark image. The container, that is, the RONI portion of the lung CT image, should be large enough to contain the watermark. The following constraint must be satisfied to proceed further in

(2) $$\begin{array}{c} {\text{Size}}_{\text{host}}\geq NP\times \text{NC}\times 8+32, \end{array}$$

where NP is the number of pixels of the watermark image and NC is the number of components (in this, NC is 3, since an RGB image has been used). After successful verification of the constraint, a header is initially inserted. The watermark image is then embedded to the bits of the host image and in the process, and the secret key is also employed. Watermark extraction is the reverse process of watermark embedding procedure. The initial input (lung image before watermarking and logo image), watermarked image, and the extracted logo are given in Figures 5(a), 5(b), 5(c), and 5(d) respectively.

## 3. Results and Discussion

The embedded, encrypted, and indexed lung CT image sent by the sender over the transmission media can face several attacks. The embedding and indexing algorithms designed are developed in such a way that the retrieving data and image has not been affected by those attacks. Of all the attacks that can be made, the following attacks were applied and evaluated.  Table 1 shows the various attacks used.

When the image is transmitted over the transmission medium, the possibility of noise to be added to that image is high. Salt and pepper noise will easily be added. As the lung CT scan images are gray scale images, an attack of altering the intensity has been applied over the image. The lens and theta values were adjusted and blurring attack performed. Unauthorized person's interruption is also considered as an attack. The embedded patient information were retrieved exactly the same as they were embedded at the sender's side. Thus, the indexing procedure provides 100% accuracy.

Unauthorized access in all forms is stopped. Since a secret key is used by the sender, unauthorized embedding into the input image before sending the image to the receiver is completely stopped. Similarly, only the receiver knowing the secret key can detect the embedded data from the retrieved image. Hence, unauthorized detection, removal, and retrieval at the receiver's side are completely blocked. Thus, it gives 100% protection against unauthorized access.

Other attacks have been performed over the images and effectiveness then verified using peak signal to noise ratio (PSNR). The unit is decibels (dB). PSNR is directly related to the accuracy of the embedding and extraction algorithms. The higher the value of PSNR, the higher the efficiency of the algorithm. The retrieved images have been used after the attack for evaluating the effectiveness of the embedding algorithm. The mean square error (MSE) is defined as [24, 25]

(3) $$\begin{array}{c} \text{MSE}=\frac{1}{mn}\overset{m}{\underset{i=1}{\sum }}\overset{n}{\underset{j=1}{\sum }}{\left[\;X\left(i,j\right)-Y\left(i,j\right)\;\right]}^{2}, \end{array}$$

where *m* and *n* are the dimensions of the images *X* and *Y*. It is calculated between the host image and the watermark embedded host image where PSNR is defined as in [24]

(4) $$\begin{array}{c} \text{PSNR}=20\cdot \log_{10}\left(\;{\text{MAX}}_{I}\;\right)-10\cdot \log_{\text{10}}\left(\;\text{MSE}\;\right), \end{array}$$

where MAX_*I*_ is the maximum possible pixel value of the image. The dataset has lung CT scan images of 23 different patients collected from Accura Scan Center, Coimbatore, India. The total number of images that were present in the dataset is 1278. The age group of the patients ranged from a minimum of 24 years to a maximum of 75 years. The number of scans for a patient varied according to the type of screening. The images present in the dataset are of 8 bit depth and of quantization level 256.

The attacks mentioned in Table 2 have been performed over the dataset of 1278 lung CT scan images and the results evaluated using PSNR. It can be predicted that it is more robust against salt and pepper noise attack than the intensity adjustment attacks. The algorithm works best if the attack is of less. PSNR value is high for attacks with less power and it gradually decreases as the power of attack increases.

Table 3 shows the average PSNR value obtained after performing attacks over the images. The watermark embedding and extraction algorithms are robust for blurring attacks with lens and theta value of 0.1 and 0.9. When the value of lens and theta is between 0.1 and 0.9, the accuracy is high and can be recovered without any noise. It is also highly secured under various attacks like noise addition and contrast reduction. The PSNR values show the robustness since the values are relatively high. The proposed method is compared with the existing approaches in Table 3. It is evident from the figure that the proposed method outperforms all the existing approaches.

## 4. Conclusion and Future Work

This paper proposed security to the image indexing process with the help of encrypted security key. The identity of the sender is verified by the receiver using the logo image retrieved with the help of the security key. Indexing images with the patient's information enhances the integrity of the images of the patient. The use of RONI is to embed the watermark which makes the medical image data untampered. From the experimental results, it is verified to be completely free from unauthorized access and blurring attacks. Other attacks like noise addition and contrast reduction affects the watermark at minimum levels. Thus, it provides a secured, authenticated, and robust way of indexing the medical images. The proposed idea shall be extended to other medical images using artificial intelligence approaches for selecting the region over which the embedding needs to be performed.

## Figures and Tables

**Algorithm 1:**
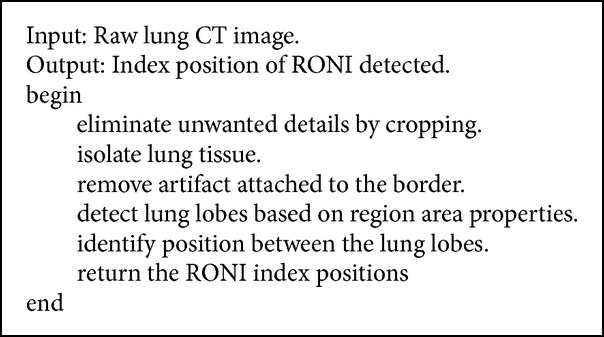
RONI detection from lung CT images.

**Algorithm 2:**
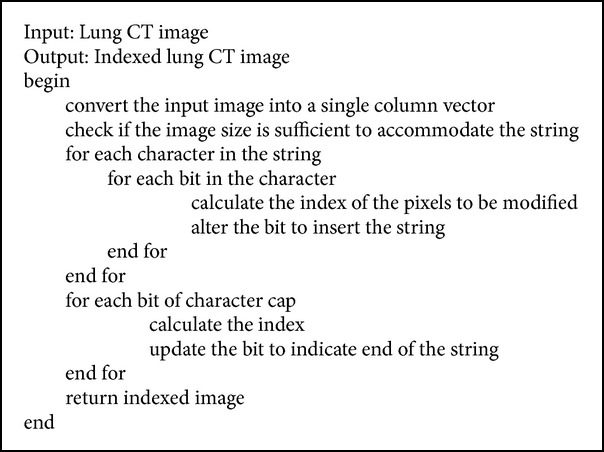
Indexing algorithm.

**Algorithm 3:**
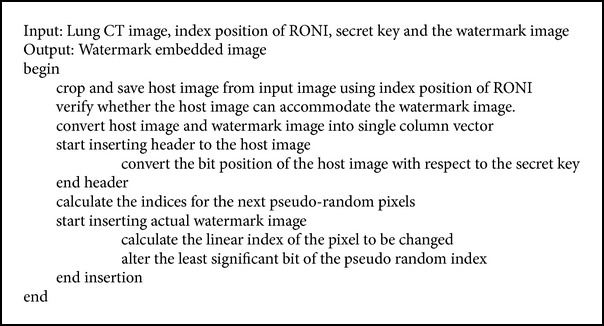
Secured watermark embedding algorithm.

**Table 1:** Attacks applied over the image.

Attacks	Variation 1	Variation 2
Noise insertion	Salt and pepper noise with 0.01 as noise density	Salt and pepper noise with 0.02 as noise density
Intensity adjustment	Reducing the contrast by 10	Reducing the contrast by 30
Blurring attack	Motion blur with lens and theta as 0.1	Motion blur with lens and theta as 0.9
Unauthorized access	Unauthorized embedding	Unauthorized detection and removal

**Table 2:** PSNR values for the corresponding attacks.

Attack	PSNR (dB)
No attack	*∞*
Salt and pepper noise with 0.01 as noise density	28.06
Salt and pepper noise with 0.02 as noise density	24.39
Reducing the contrast by 10	23.19
Reducing the contrast by 30	21.89
Motion blur with lens and theta as 0.1	*∞*
Motion blur with lens and theta as 0.9	*∞*

**Table 3:** PSNR values for the existing and proposed methods.

Method used (without attack)	PSNR (dB)
Discrete cosine transform (DCT) based watermark embedding [26]	32.34
Local binary pattern (LBP) based watermark embedding [27]	39.79
Integer wavelet transform (IWT) based watermark embedding [28]	29.67
Watermarking based on redundant wavelet transform and singular value decomposition (RDWT-SVD) based watermark embedding [25]	36.69
Proposed: RONI based secured and authenticated indexing	*∞*
